# Improved Detection of Magnetic Signals by a MEMS Sensor Using Stochastic Resonance

**DOI:** 10.1371/journal.pone.0109534

**Published:** 2014-10-15

**Authors:** Agustín L. Herrera-May, Jesus A. Tapia, Saúl M. Domínguez-Nicolás, Raul Juarez-Aguirre, Edmundo A. Gutierrez-D, Amira Flores, Eduard Figueras, Elias Manjarrez

**Affiliations:** 1 Micro and Nanotechnology Research Center, Universidad Veracruzana, Boca del Río, Veracruz, México; 2 Institute of Physiology, Benemérita Universidad Autónoma de Puebla, Puebla, Puebla, México; 3 School of Biology, Benemérita Universidad Autónoma de Puebla, Puebla, Puebla, México; 4 Department of Electronics, Instituto Nacional de Astrofísica Óptica y Electrónica, INAOE, Puebla, Puebla, México; 5 Department of Automatic Control, CINVESTAV-IPN, Mexico City, Distrito Federal, México; 6 Microelectronics Institute of Barcelona, IMB-CNM, CSIC, Bellaterra, Barcelona, Spain; Wake Forest University School of Medicine, United States of America

## Abstract

We introduce the behavior of the electrical output response of a magnetic field sensor based on microelectromechanical systems (MEMS) technology under different levels of controlled magnetic noise. We explored whether a particular level of magnetic noise applied on the vicinity of the MEMS sensor can improve the detection of subthreshold magnetic fields. We examined the increase in the signal-to-noise ratio (SNR) of such detected magnetic fields as a function of the magnetic noise intensity. The data disclosed an inverted U-like graph between the SNR and the applied magnetic noise. This finding shows that the application of an intermediate level of noise in the environment of a MEMS magnetic field sensor improves its detection capability of subthreshold signals via the stochastic resonance phenomenon.

## Introduction

Stochastic resonance is a phenomenon of nonlinear systems characterized by a response increase of the system induced by a particular level of input noise. The essential feature of this phenomenon is that the SNR versus input noise is an inverted U-like function characterized by maximal enhancement of SNR at a specific noise intensity value.

In biology, Douglass et al. [1] published the first description of stochastic resonance (SR) in crayfish mechanoreceptors. Further studies involved the analysis of SR in other sensory receptors for tactile, vestibular, auditory and visual modalities [2]–[16]. These biological studies sparked alternative theoretical approaches and the development of new sensors that employ noise to improve their detection capability [17]–[24]. For instance, arrays of MEMS flow sensors [24] were inspired by the acoustic flow-sensitive hairs of the cricket's cerci.

Although there are diverse biological and artificial sensors employing noise to improve signal detection, there are not yet fabricated sensors that include magnetic noise to detect weak magnetic fields. Moreover, there is little information about the phenomenon of stochastic resonance associated to magnetic fields. The first formal description of such phenomenon was introduced by Grigorenko et al., [25]–[26], who proposed a method for magnetic field measurement in nanometer scale. This method is based upon stochastic resonance in arrays of magnetic nanoparticles. The magnetostochastic resonance was proposed for studying magnetization fluctuations in a ferromagnet, in particular, for observing a macroscopic quantum tunneling of the magnetic moment [25]. Subsequently, in 1995 Hibbs et al. [27] described an experiment in which the magnetic signal detected by a radio frequency superconducting quantum interference device (RF SQUID) sensor was enhanced by the addition of an optimal level of noise into the device. However, these studies did not report the development of a magnetic field sensor including a magnetic noise generator to detect weak magnetic fields. The purpose of the present study is to introduce the first MEMS magnetic field sensor that can improve the detection of subthreshold magnetic field signals by applying magnetic noise. In this case, the magnetic noise is injected in the vicinity of a simpler sensor based on microelectromechanical systems (MEMS) technology.

We employed the same MEMS sensor as in our previous studies [28]–[37], [39], [40]–[41]. The MEMS sensors have potential applications in automotive industry, military instruments, telecommunications, and the biomedical sector [38]–[41]. MEMS sensors have important advantages such as a small size, a lightweight, low-power consumption, and a high resolution [38]. Most of these sensors make use of the Lorentz force to detect a magnetic field through the use of different sensing techniques, including the capacitive, the optical, or the piezoresistive. The importance of the present study is that these sensors could improve their detection capabilities for subthreshold magnetic fields using an intermediate level of magnetic noise.

## Materials and Methods

### MEMS magnetic field sensor

**Figure 1:**
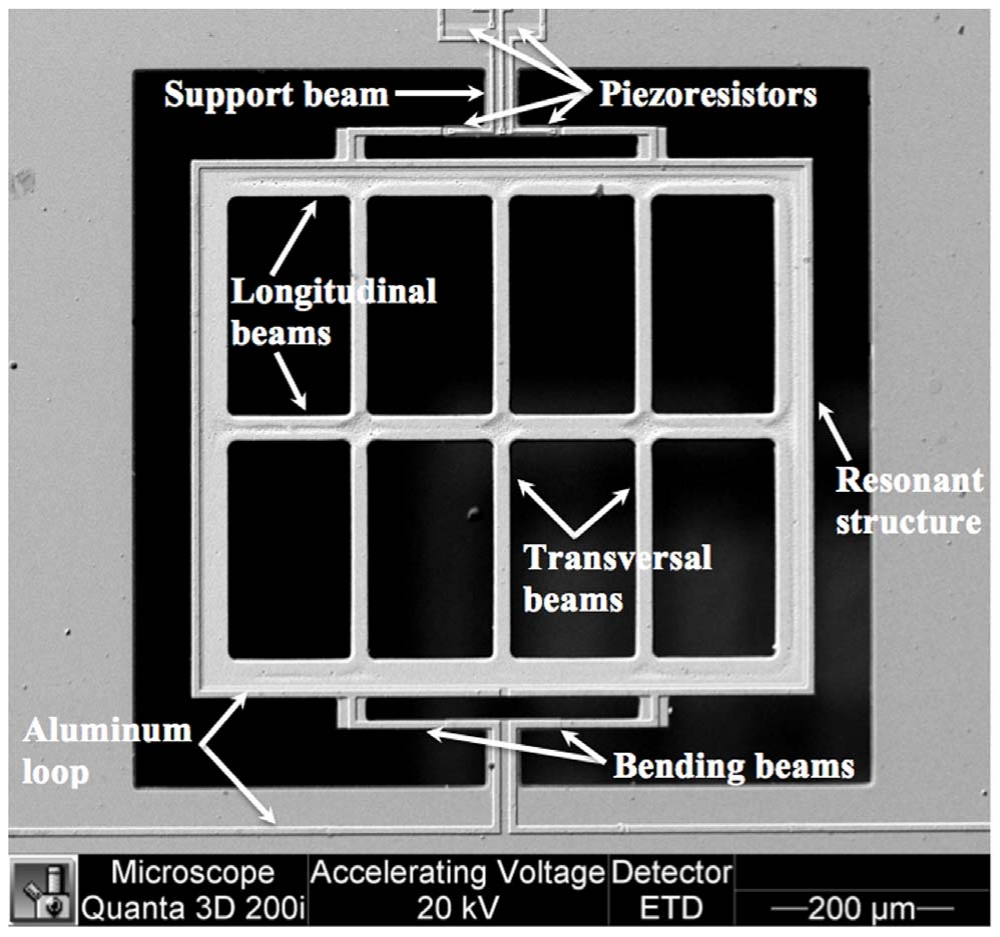
SEM image of the resonant structure of the MEMS magnetic field sensor.

The MEMS magnetic field sensor makes use of the Lorentz force based on a piezoresistive sensing technique. This sensor includes a resonant silicon structure (700 µm×600 µm×5 µm), an aluminum loop (1 µm thickness) and a Wheatstone bridge with four type-p piezoresistors, as shown in Figure 1. It has been developed by the MEMS group from the Micro and Nanotechnology Research Center (MICRONA) of the Universidad Veracruzana with collaboration of the Microelectronics Institute of Barcelona (IMB-CNM, CSIC) [13]. Its resonant structure consists of four bending silicon beams and an arrangement of longitudinal and transversal silicon beams. This resonant structure is connected to a silicon substrate by means of two support beams (60×40×5 µm). In addition, two piezoresistors are placed on the surface of the silicon substrate and other two piezoresistors are located on two bending beams.

**Figure 2:**
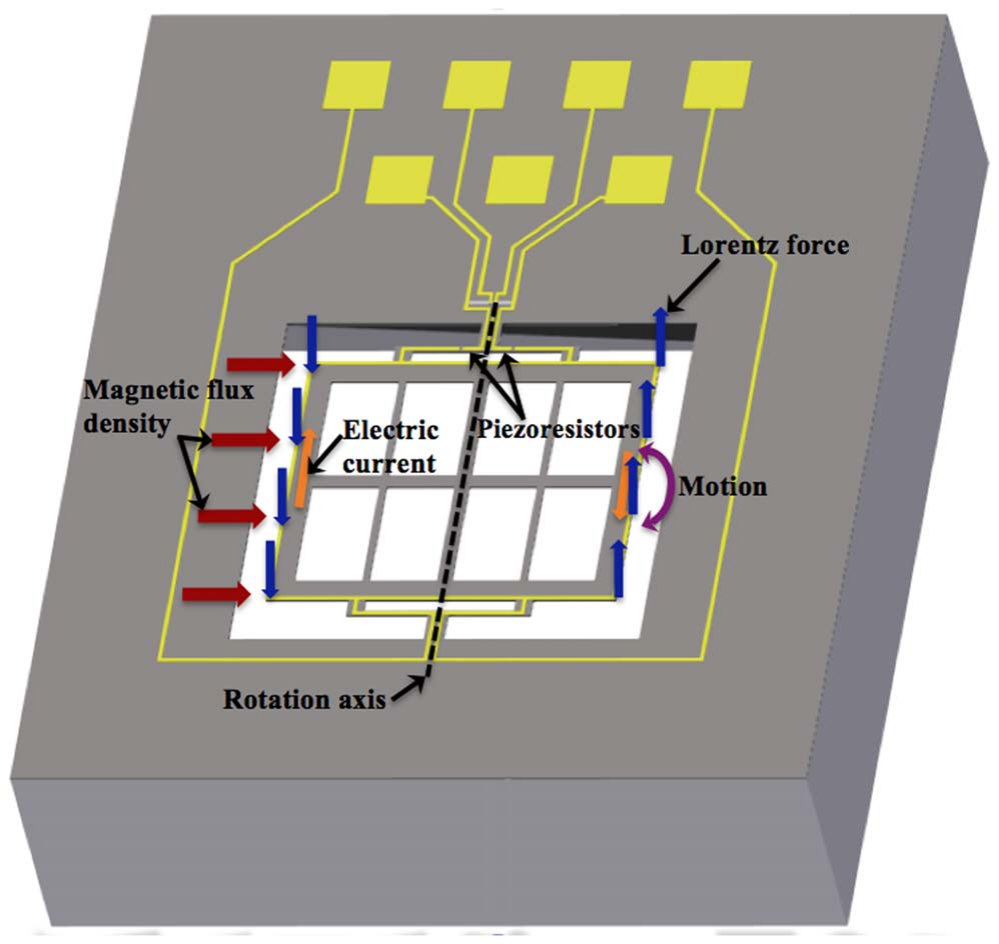
Schematic view of the operation principle of the MEMS magnetic field sensor.

A sinusoidal electrical current is applied through the aluminum loop of the MEMS sensor to interact with an external magnetic flux density parallel to the length of the resonant structure. This interaction generates a Lorentz force on the structure, which causes an oscillation motion (see Figure 2). Then it is amplified when the frequency of the electrical current is equal to the first bending resonant frequency of the MEMS sensor structure. Due to this amplified motion, the piezoresistors located on two bending beams are subjected to a longitudinal strain that changes their initial no-strain resistances. It produces a change in the output voltage of the Wheatstone bridge. Thus, the electrical signal of the MEMS sensor is related to the applied magnetic flux density.

### Design of the signal conditioning system and virtual instrument

**Figure 3:**
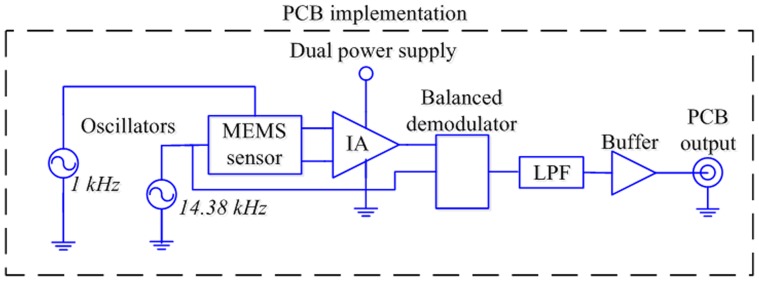
Block diagram of the signal conditioning system of the MEMS magnetic field sensor.

We designed a signal conditioning system implemented on a PCB for the MEMS sensor, which contains oscillators with high-frequency stability around ±100 ppm at room temperature. In order to excite the sensor in its first resonant frequency, we made an algorithm in a digital signal controller dsPIC30F4013 (Microchip Technology Inc) to assure a frequency sweep with a resolution of 1 Hz. This system allows the approximately linear measurement of the polarity and magnitude of magnetic flux density with a minimum offset. The Figure 3 shows the block diagram of the signal conditioning system for the MEMS sensor.

### Experimental setup and emitted magnetic field signals

**Figure 4:**
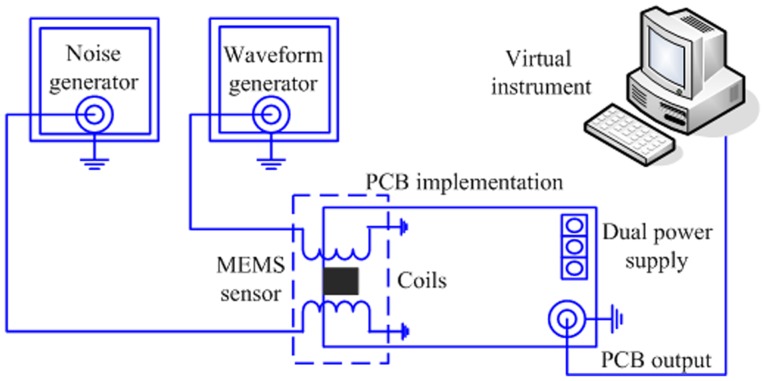
Experimental setup for the detection of magnetic signals by a MEMS sensor using stochastic resonance.

Pulsed and noisy magnetic fields were generated by means of two miniature solenoids with 250 loops of insulated AWG-24 cooper wire. The experimental setup is shown in the Figure 4.

**Figure 5:**
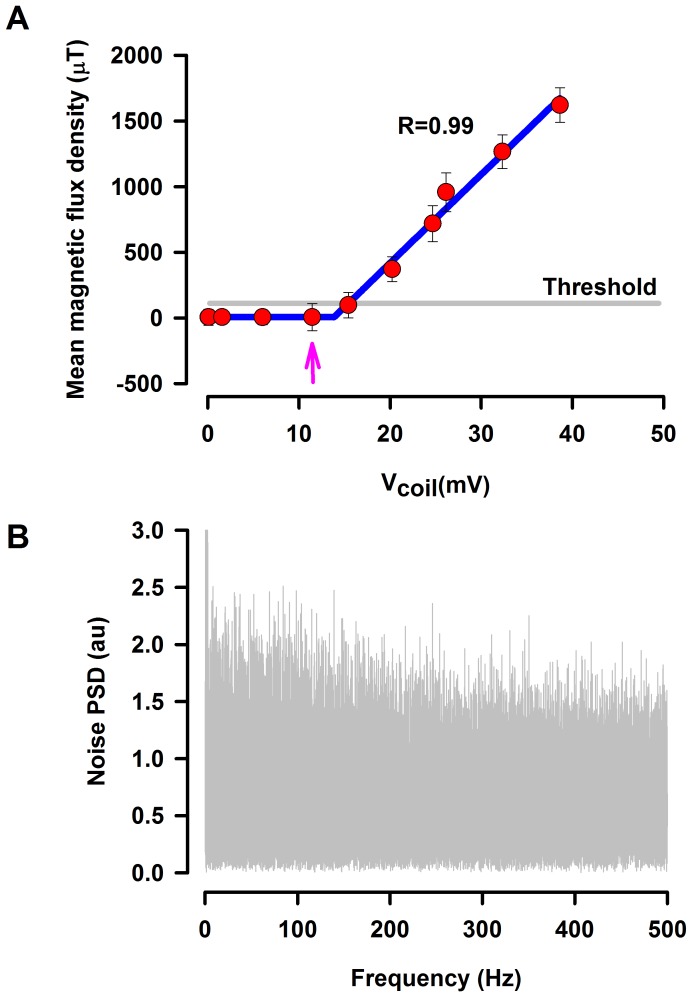
Graph employed to determine the threshold of our MEMS sensor and a graph illustrating the typical input noise. **A**, input-output curve to identify the threshold level of detection of our MEMS sensor. The horizontal axis indicates the input signal; i.e., the input voltage applied in the coil. The vertical axis shows the output signal; i.e., the magnetic flux density detected with the MEMS sensor. This type of input-output curve was useful to select the appropriated subthreshold signal (see vertical arrow) for every stochastic resonance experiment. **B**, the typical power spectra density (PSD) of magnetic noise (Gaussian noise from 0 to 500 Hz).

The PCB of the MEMS sensor is energized with a dual power supply Agilent E3631A. A Master-8 waveform generator (AMPI, Jerusalem) was used to produce the test signal, which was applied to the first coil. This coil is placed in the region where the MEMS sensor detects the largest magnetic field. Furthermore, a second coil is employed to produce the magnetic noise (white Gaussian noise, from 0 to 500 Hz) by means of a Wavetek noise generator (Model 132, San Diego, CA, USA). The typical power spectrum of this magnetic noise is illustrated in Figure 5B.

The PCB output represents the MEMS sensor response in voltage mode. This signal is processed with the designed virtual instrument. The data acquisition in voltage mode is feed through the PCI-DAS6031 card (Measuring Computing Corporation).

### Subthreshold magnetic signals

We examined the effects of magnetic noise on the detection capability of our MEMS sensor to detect subthreshold magnetic signals. These subthreshold signals were generated by a coil and consisted of pulsed magnetic signals of 100 ms elicited every second (i.e., at 1 Hz) during 120 seconds.

### Data analysis

Data acquisition of the magnetic signal and magnetic noise was performed with a sampling rate of 300 kHz (Digidata 1400 A, Axon Instruments, Molecular Devices). Spectral analysis of the detected magnetic signals with the MEMS sensor was performed. The magnitude of the input magnetic noise was quantified by means of the standard deviation of the input noise. We employed the signal-to-noise ratio (SNR) to estimate the effect of magnetic noise upon the capability of the MEMS sensor to detect subthreshold magnetic signals. We computed the signal-to-noise ratio (SNR) for our experimental data as in previous studies about stochastic resonance from our laboratory [10], [12].

We defined the SNR as the ratio, at the input signal frequency (1 Hz), of the strength of the output power spectra peak (its area) during pulse stimulation plus noise to the output power spectra area occurring during input noise alone. Both areas were calculated in the frequency interval of ±0.1 Hz around the input signal frequency (1 Hz). The method to calculate SNR was the following:
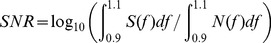
(1)


*S(f)* corresponds to the power spectrum of the periodic magnetic signal detected with the MEMS sensor plus magnetic noise. *N(f)* is the power spectrum of the magnetic noise alone detected with the same MEMS sensor.

### Statistical analysis of SNR

Data were expressed as mean ± sd. The statistical difference in SNR between zero noise and optimal noise was determined by the Wilcoxon test. The comparison was considered to be significant if p<0.05.

## Results

We examined the effects of magnetic noise on the detection capability of our MEMS sensor to detect subthreshold magnetic signals. First, we obtained the input-output graph (Figure 5A, Table 1) to characterize our MEMS sensor (i.e., input is the voltage applied in the coil and output is the magnetic flux density detected with the MEMS sensor). Second, we also applied subthreshold magnetic signals to identify the threshold level of detection capability of our MEMS sensor. Third, we selected the intensity of the magnetic flux density that was just below the threshold level of detection of our MEMS sensor (see arrow in Figure 5A). Fourth, this subthreshold signal was employed to examine the effects of seven different levels of magnetic noise on the detection capability of our MEMS sensor to detect this subthreshold signal.

**Table 1:** MEMS magnetic flux density measured to calculate the detection threshold of the MEMS sensor (See figure 5A).

V_COIL_ (mV)	MEMS Magnetic Flux Density (µT)					MEAN (µT)	STD (µT)
	EXP 1	EXP 2	EXP 3	EXP 4	EXP 5		
**0.1**	−26.1	12.9	8.2	95.9	−60.6	**6.1**	**58.3**
**0.1**	−33.8	36.4	57.0	7.2	−38.5	**5.6**	**42.1**
**1.6**	33.9	−49.4	38.2	−26.0	47.1	**8.8**	**43.5**
**6.0**	−47.6	47.4	−27.9	82.4	−40.5	**2.8**	**58.5**
**11.4**	−62.3	21.5	91.8	−131.6	115.2	**6.9**	**103.8**
**15.4**	78.2	186.6	−56.2	169.3	104.2	**96.4**	**96.3**
**20.2**	326.4	424.1	396.2	234.3	470.7	**370.3**	**92.3**
**24.7**	900.3	563.9	613.9	704.0	823.1	**721.0**	**140.5**
**26.1**	1145.9	978.0	952.7	962.3	736.6	**955.1**	**145.6**
**32.3**	1074.0	1212.0	1319.8	1392.4	1336.3	**1266.9**	**126.1**
**38.6**	1687.1	1473.1	1805.6	1631.6	1513.7	**1622.2**	**134.1**

We explored whether a particular level of magnetic noise applied on the vicinity of the MEMS sensor can improve the detection of subthreshold magnetic fields. As described in methods section we employed two coils, one to emit the magnetic signal and other to emit the magnetic noise. The MEMS sensor was placed in the region of the maximal magnetic flux density signal emitted by a coil.

**Figure 6:**
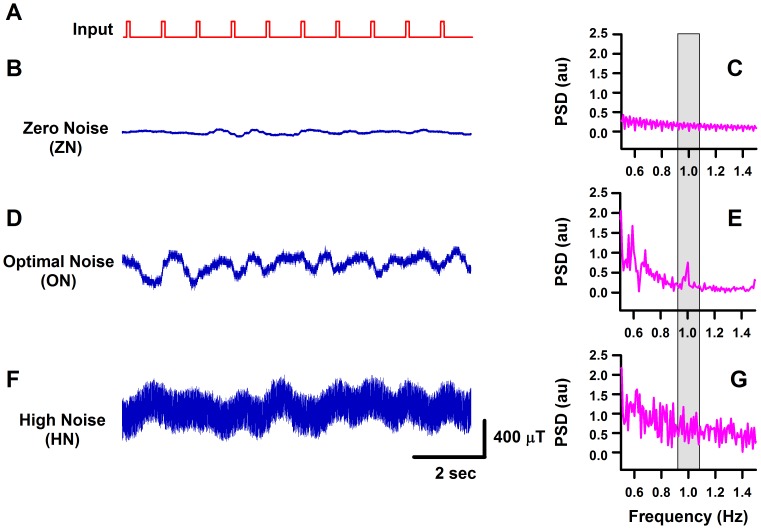
Recordings of continuous MEMS magnetic flux density and their corresponding power spectrum density. **A**, applied subthreshold magnetic stimulus (input). **B, D and F**, recordings of the detected MEMS magnetic flux density at three noise levels: zero, optimal, and high noise. Note that the probability to detect a signal is increased when an optimal level of magnetic noise was added. **C, E and G**, corresponding power spectrum densities (PSD) for the MEMS recordings illustrated in the left panel. The power spectra of the MEMS show a peak at the input frequency (1 Hz) for optimal noise but not for zero or high noise. The gray rectangle in the PSD illustrates the frequency of the detected periodic magnetic signal in which there is a peak for the optimal noise level.

We observed the stochastic resonance phenomenon in 5 of 5 experiments. We found that the application of an intermediate level of noise in the environment of the MEMS magnetic field sensor improves its capability to detect subthreshold magnetic signals. Figures 6B and 6C show a magnetic signal below the threshold and the power spectrum, respectively. Note that the power spectrum does not exhibit a peak at the input frequency (1 Hz); i.e., the subthreshold magnetic signal was not detected by our MEMS magnetic sensor at the zero noise level. However, our MEMS sensor was able to detect such subthreshold signal when an optimum level of magnetic noise was applied (see Figures 6D and 6E). Note the peak at 1 Hz in the power spectrum of Figure 6E. Moreover, our MEMS sensor was unable to detect the subthreshold signal when a high level of noise was applied (Figures 6F and 6G).

**Figure 7:**
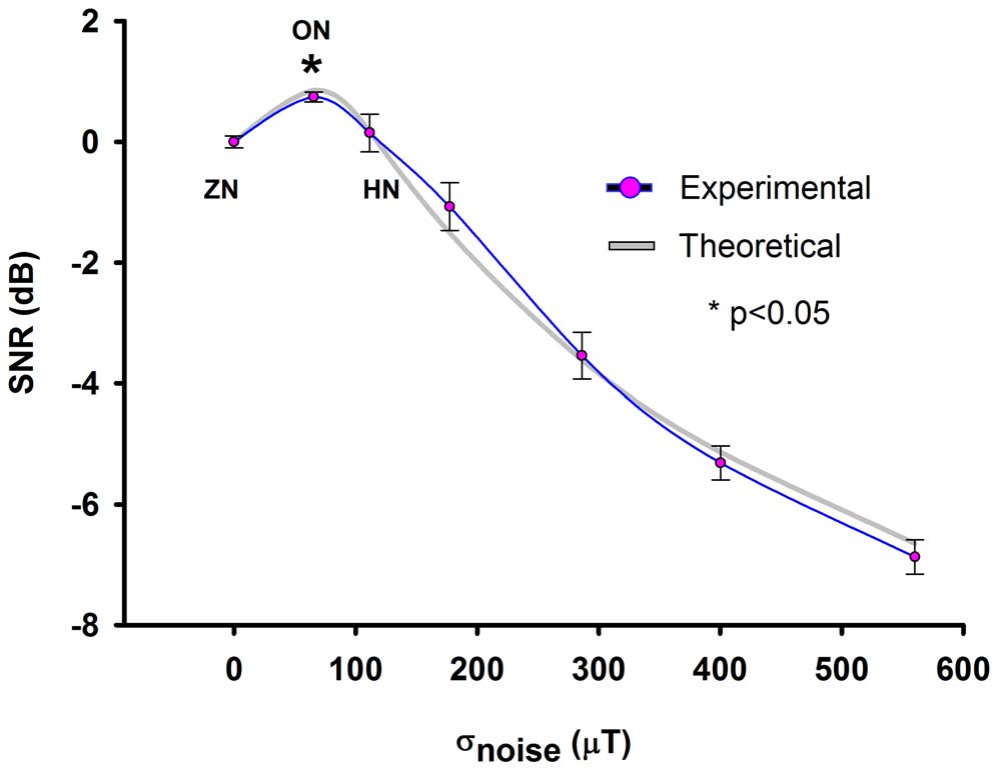
Experimental vs Theoretical SNR. The magenta circles show pooled data from five experiments. The blue line is the signal-to-noise ratio (SNR) computed from our experiments, while the gray line is the theoretical SNR obtained with the Moss-equation (Moss et al., [43]–[44]; parameters: *A* = 0.1717; *ω_n_* = 5000; *Δ_0_* = 10.66). The addition of an optimal noise level increases the SNR and the probability of detecting a subthreshold magnetic signal from the miniature coil. The optimal magnetic noise that improves the capability detection of the MEMS sensor is indicated as ON. Zero noise (ZN), high noise (HN). Values above 100 µT represent HN. The asterisk indicates statistically significant difference for the SNR between optimal noise and zero noise conditions (p<0.05, Wilcoxon test).

In Figure 7 we show pooled data of the SNR versus different levels of noise (Table 2). We found that there is a statistically significant intermediate-non-zero level of noise that improves the capability of our MEMS sensor to detect subthreshold magnetic signals. In Figure 7 we also show a theoretical curve to compare our experimental results with the theory of stochastic resonance of a nonlinear system with a bistable behavior. Moss et al. [43]–[44] suggested an equation (2) for the characteristic SNR associated with the stochastic resonance. This equation requires the amplitude of the periodic signal (*A*), the cutoff frequency (*ω_n_*) and the difference between the subthreshold signal and the threshold (*Δ_0_*). The equation reads as follows:
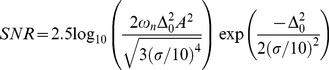
(2)


**Table 2:** SNR calculations from 5 experiments in which we applied several increasing input noises (See Figure 7).

NOISE STD (µT)	SNR (dB)					MEAN SNR (dB)	STD SNR (dB)
	EXP 1	EXP 2	EXP 3	EXP 4	EXP 5		
**0.00**	−0.09	−0.05	0.01	0.29	−0.17	**0.00**	**0.18**
**65.33**	0.69	0.82	0.62	0.76	0.78	**0.74**	**0.08**
**111.53**	0.15	0.05	−0.30	0.20	0.36	**0.09**	**0.25**
**177.39**	−0.44	−1.47	−1.27	−1.14	−1.02	−**1.07**	**0.39**
**286.19**	−3.69	−2.87	−3.15	−3.46	−3.56	−**3.35**	**0.33**
**400.39**	−5.15	−5.39	−5.59	−4.98	−5.43	−**5.31**	**0.24**
**560.22**	−7.04	−6.78	−6.90	−7.06	−6.45	−**6.85**	**0.25**

This equation qualitatively fits to the described SNR for the stochastic resonance phenomena. The theoretical parameters that we employed to fit our experimental results are: *A* = 0.1717; *ω_n_* = 5000; *Δ_0_* = 10.66. The addition of an optimal noise level increases the detection probability of a magnetic stimulus from the miniature coil.

## Discussion

We demonstrated that an intermediate level of magnetic noise applied on the vicinity of our MEMS magnetic field sensor could help to improve the detection of subthreshold magnetic fields using stochastic resonance. We suggest that this physical phenomenon could be employed to increase the output voltage signal of MEMS devices with piezoresistive sensing. These MEMS sensors subjected to a determined magnetic noise could increase its dynamic range and sensitivity without the need of using a vacuum package or any change in the operation parameters of the MEMS sensor.

The aim of our article was to demonstrate the stochastic resonance phenomenon associated with our MEMS sensor. In this context, we demonstrated that sub-threshold magnetic signals (below the detection limits) can be detected by a MEMS sensor when an optimal level of magnetic noise is applied. The relative enhancement of 12% is enough to demonstrate the stochastic resonance phenomenon because we obtained a statistically significant difference between the zero noise and the optimal noise condition (* p<0.05 Wilcoxon test, in Figure 7). With this statistically significant difference we are compellingly demonstrating the occurrence of such phenomenon in our system. Future improvements in the technology of the MEMS magnetic field sensors could help to improve the detection of magnetic fields above the 12% of enhancement. Moreover, in Figure 7 we also show a theoretical curve (base on the well accepted stochastic resonance equation) which compellingly fits with our experimental results.

In order to clarify why the output of the MEMS device is a RC-like signal and not a square wave signal as the input signal in the coil, we provide the following explanation. The MEMS sensor consists of a resonant silicon structure, an aluminum loop, and a Wheatstone bridge of four type-p piezoresistors. This sensor operates with the Lorentz force, which is proportional to the interaction between a sinusoidal excitation current of 20 mA at 14.376 kHz and an external magnetic field. This magnetic field is caused by square wave signals with a delay time, which is generated by an excitation coil. Thus, the generated Lorentz-force has signal form that is the result of the interaction between a sinusoidal signal and square wave signal with a delay time. During this delay time, the Lorentz-force response has a minimum magnitude. This Lorentz force causes a deformation of piezoresistors located on two flexural beams, which alters their initial resistance values. It generates an output-voltage shift of a Wheatstone bridge (electrical response of the MEMS sensor), which is supplied by an input voltage of 1 Vp at 1 kHz. Therefore, this electrical response has a signal form resulted of the combination between the Lorentz force signal and the input voltage signal. After, the electrical response of MEMS sensor is filtered using an arrangement of low-pass RC Filters, which causes a RC-like signal instead of a square wave signal.

In the current study we employed the same noise generator [10]–[13], [16], as well as the same MEMS magnetic field sensor [40]–[41] described in our previous reports in the biomedical research and in the development of MEMS technology. Recently, we used such MEMS sensor to detect the respiratory magnetogram [40]. Such sensor has a magnetic detection-sensitivity in the range of a few hundreds of nanoTesla. It is tempting to suggest that the addition of optimal magnetic noise to our MEMS sensor could also be useful to increase its detection capability of subthreshold respiratory magnetograms.

The graph illustrated in Figure 7 suggests that the MEMS sensor exhibits the stochastic resonance, a counterintuitive phenomenon in which an intermediate level of noise improves the detection of a weak signal. Many nonlinear sensors exhibit this phenomenon [19]–[20]. We suggest that the nonlinear properties of our MEMS sensor can contribute to this peculiar behavior, in which an intermediate level of noise improves its detection capabilities. The SNR increase of the magnetic flux density detected by the MEMS sensor versus several levels of magnetic noise (Figure 7) could be explained by a threshold system with a nonlinear bistable behavior, which is an intrinsic property of many sensors, including the biological sensors (see references [42]–[44] for the SR model and equation (2)).

The stochastic resonance applied to MEMS magnetic field sensors helps to enhance its magnetic sensitivity and therefore expand its potential applications. For instance, these sensors could be used to detect cracks and zones of stress concentration of ferromagnetic structures using the metal magnetic memory method [45]–[46]. Another application could be the detection of magnetically marked diagnostic capsules in real time inside human body [45], [47]–[48]).

The fabrication of miniature and encapsulated MEMS magnetic field sensors should include stochastic-resonance coils on the same chip. These coils could be designed with metallic materials deposited on the silicon substrate, around the active area of the MEMS sensor. They could be supplied with an intermediate level of electrical current noise, which would generate a magnetic noise applied on the MEMS sensor. This noise could increase the dynamic range and sensitivity of the MEMS sensor, thus allowing the operation of the modified MEMS sensors in noisy environments, which could be very useful for medical applications and sensing of neuronal magnetic fields.

Specifically, in the sensor design process we could include two integrated planar coils around the sensor to generate an auxiliary magnetic field that operates as magnetic noise. It could increase the electrical response of the magnetic field sensor to detect weak magnetic fields. These planar coils could be fabricated on the same silicon wafer using a physical vapor deposition (PVD) of aluminum [32], [49]. Each coil could generate a magnetic noise using electrical current pulses with different amplitudes (about 100 mA) and random frequencies. For this, the sensor-design phase must include the modeling of the planar coils to obtain their optimal dimensions and geometrical configuration, which will depend of the sensor design. Thus, the coils design must guarantee the generation of a magnetic noise on the sensor, keeping a measurement system with small size, low consumption power, and high resolution. For instance, two integrated planar coils can be designed using aluminum windings with a linewidth and thickness of 50 µm and 1 µm, respectively.

Finally, an advantage potential of the magnetic stochastic resonance in the performance of a resonant magnetic field sensor is the capacity for monitoring weak magnetic field close to the sensor resolution. For instance, weak magnetic fields related with cracks or zones of stress concentration in ferromagnetic structures [45]–[46] could be detected using magnetic field sensors with integrated planar coils that generate a magnetic stochastic resonance.
